# Supplementary material for: Few-Shot Learning Enables Population-Scale Analysis of Leaf Traits in Populus trichocarpa
Source: Plant Phenomics. 2023 Jul 28;5:0072. doi: 10.34133/plantphenomics.0072 (PMC10380552; doi:10.34133/plantphenomics.0072)
Supplement: Supplementary 1 — Figs. S1 and S2 Tables S1 to S3 Movies S1 and S2 [file plantphenomics.0072.f1.zip › SM.pdf]

Supplementary Materials

Video S1: Leaf segmentation video. Animation of the leaf tracing algorithm, in which a CNN iteratively traces the boundary of a leaf. Left: the raw leaf scan with an overlay of the previously traced path and a bounding box indicating the current position of the CNN model. Top right: the image tile and with an overlay of the previously traced path that are input to the CNN. Bottom right: the predicted pixels along the contour of the leaf that are used to update the position in the next iteration. The iteration proceeds until the CNN predictions reach the start of the trace. Note that in practice the iteration completes in $\sim$1 second, but is slowed down for better visualization.

Video S2: Vein segmentation video. Animation of the vein growing algorithm, in which a CNN iteratively adds pixels to a growing segmentation of the visible vein architecture. Left: the original leaf scan with an overlay of the pixels being considered by the CNN in yellow and classified vein pixels in red. Top right: a zoomed in view of the top of the leaf and overlay. Bottom right: a zoomed in view of the bottom of the leaf and overlay. The iteration proceeds, continuously adding new pixels to the segmentation, until no new pixels remain in the sample set. Note that in practice the iteration completes in $\sim$60 seconds, but is sped up for better visualization.

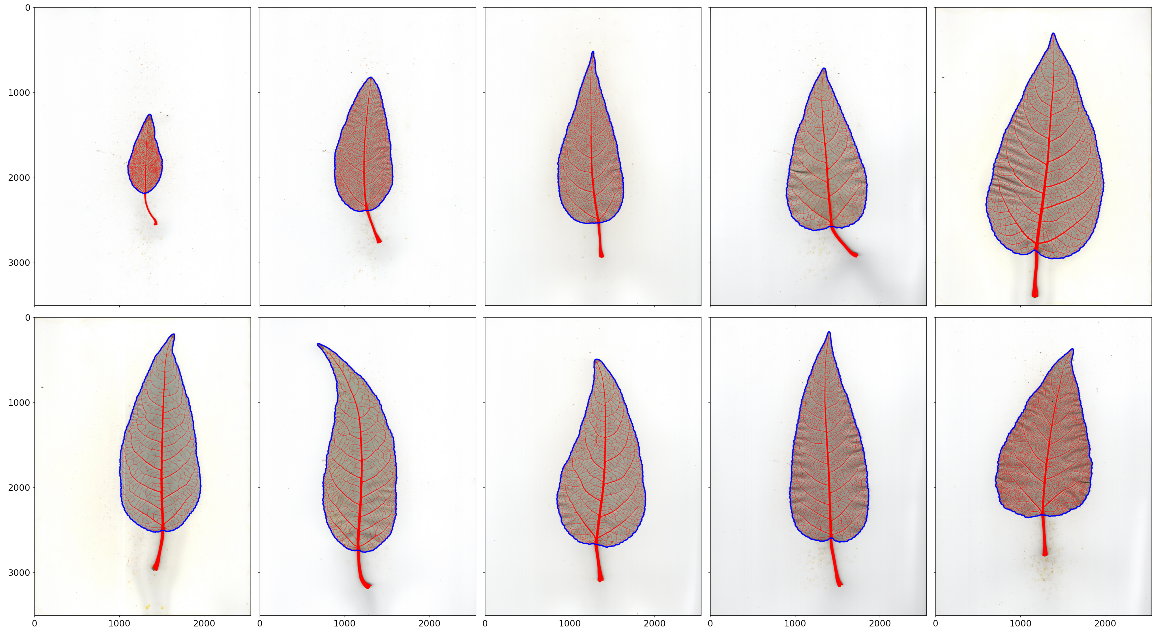

Figure S1: Example leaf and vein segmentations. Results of the leaf and vein segmentation methods on example leaf images outside the training set. Traced leaf contours are shown in blue and vein segmentations in red. Top: segmentation overlays for leaves varying in size, going from smallest (left) to largest (right). Bottom: segmentation overlays for leaves of approximately equal area, but varying in vein density, going from sparse (left) to dense (right) venation.

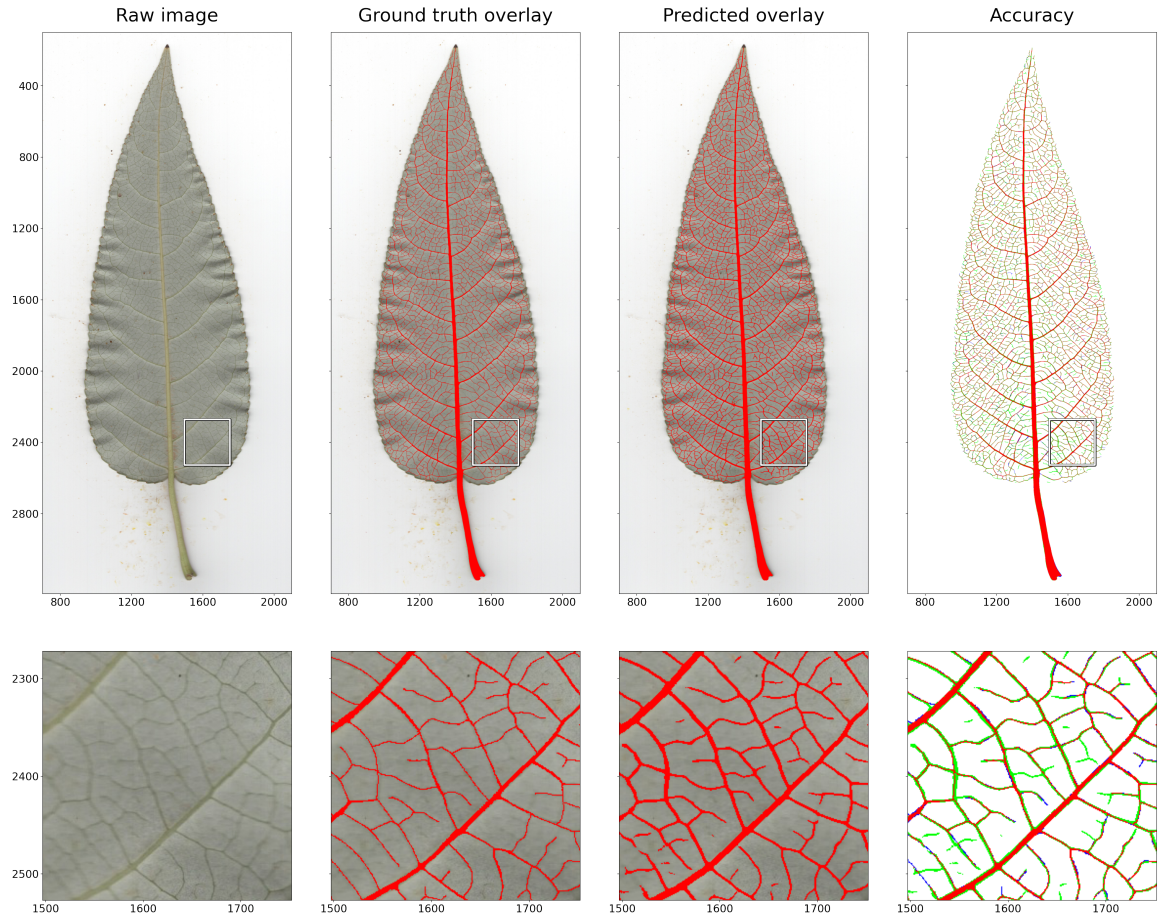

Figure S2: Vein segmentation accuracy. Results of the vein segmentation method on a leaf from the validation set. The top row shows the full leaf and the bottom row gives a zoomed in view. Left: example leaf scan chosen from the validation set. Center left: hand-annotated vein segmentation overlaid in red. Center right: predicted vein segmentation overlaid in red. Right: a comparison between the ground truth and predicted segmentations, in which red pixels indicate true positives, green pixels indicate false positives, and blue pixels indicate false negatives. Note that the zoomed-in tile reveals veins identified by the region growing method that are incorrectly reported as false positives (see veins with only green pixels) due to errors in the ground truth segmentation.

Table S1: Leaf features. Includes names, units, broad-sense clonal heritability estimates, and descriptions of the 23 traits related to leaf morphology and color. Abbreviations: avg: average, max: maximum, min: minimum.

Feature	Units	H^2	Tool	Description
Area	cm^2	0.30	Fiji	Total pixel count of leaf segmentation
Aspect ratio	-	0.58	Fiji	Ellipse major axis / ellipse minor axis
Bottom blue	-	0.57	Fiji	Avg. blue value of leaf abaxial side
Bottom brightness	-	0.42	Fiji	Avg. brightness value of leaf abaxial side
Bottom green	-	0.41	Fiji	Avg. green value of leaf abaxial side
Bottom hue	-	0.39	Fiji	Avg. hue value of leaf abaxial side
Bottom red	-	0.45	Fiji	Avg. red value of leaf abaxial side
Bottom saturation	-	0.27	Fiji	Avg. saturation value of leaf abaxial side
Circularity	-	0.23	Fiji	$4\pi A/P^2$ where A: area and P: perimeter
Convex area	mm^2	0.29	RVE	Total pixel count of convex hull
Major axis length	cm	0.21	Fiji	Major axis length of best-fit ellipse
Minor axis length	cm	0.44	Fiji	Minor axis length of best-fit ellipse
Max. Feret	cm	0.23	Fiji	Max. distance between any two points in the leaf segmentation
Min. Feret	cm	0.42	Fiji	Min. distance between two parallel lines tangent to Max. Feret line
Perimeter	cm	0.25	Fiji	Sum of Euclidean distances between contour pixels in the leaf segmentation
Roundness	-	0.56	Fiji	$4A/(\pi M^2)$ where A: area, M: major axis
Solidity	-	0.09	Fiji	A/C where A: area and C: convex area
Top blue	-	0.26	Fiji	Avg. blue value of leaf adaxial side
Top brightness	-	0.26	Fiji	Avg. brightness value of leaf adaxial side
Top green	-	0.23	Fiji	Avg. green value of leaf adaxial side
Top hue	-	0.29	Fiji	Avg. hue value of leaf adaxial side
Top red	-	0.24	Fiji	Avg. red value of leaf adaxial side
Top saturation	-	0.21	Fiji	Avg. saturation value of leaf adaxial side

Table S2: Vein features Includes names, units, broad-sense clonal heritability estimates, and descriptions of the 27 traits related to vein morphology. Abbreviations: avg: average, DR: diameter range, max: maximum, min: minimum, RVE: RhizoVision Explorer.

**Feature**	**Units**	H^2	**Tool**	**Description**
Area	mm^2	0.43	RVE	Total pixel count of vein segmentation
Area DR 1	mm^2	0.55	RVE	Projected area of veins with DR 0 - 0.25 mm
Area DR 2	mm^2	0.38	RVE	Projected area of veins with DR 0.25 - 0.8 mm
Area DR 3	mm^2	0.26	RVE	Projected area of veins with DR above 0.8 mm
Avg. diameter	mm	0.34	RVE	Avg. skeletal pixel radius, doubled for diameter
Convex area	mm^2	0.29	RVE	Total pixel count of convex hull
Density	-	0.65	Custom	Ratio of vein area to leaf area
Length-to-area ratio	-	0.62	RVE	V/A where V: total length, A: leaf area
Max. depth	mm	0.24	RVE	Max. vertical distance in vein segmentation
Max. diameter	mm	0.32	RVE	Max. skeletal pixel radius, doubled for diameter
Max. width	mm	0.41	RVE	Max. horizontal distance in vein segmentation
Network solidity	-	0.64	RVE	Network Area per Convex Area ratio
Perimeter	mm	0.52	RVE	Sum of Euclidean distances between contour pixels in the vein segmentation
Surface area	mm^2	0.46	RVE	Length multiplied by cross-section circumference summed over skeletal pixels.
Surface area DR 1	mm^2	0.55	RVE	Surface area of veins with DR 0 - 0.25 mm
Surface area DR 2	mm^2	0.38	RVE	Surface area of veins with DR 0.25 - 0.8 mm
Surface area DR 3	mm^2	0.26	RVE	Surface area of veins with DR above 0.8 mm
Third order fraction	-	0.29	RVE	Ratio of total length of DR 3 to total length
Total length	mm	0.53	RVE	Sum of Euclidean distances between connected skeletal pixels
Total length DR 1	mm	0.56	RVE	Total length of veins with DR 0 - 0.25 mm
Total length DR 2	mm	0.40	RVE	Total length of veins with DR 0.25 - 0.8 mm
Total length DR 3	mm	0.27	RVE	Total length of veins with DR above 0.8 mm
Volume	mm^3	0.29	RVE	Length multiplied by cross-section area summed over skeletal pixels
Volume DR 1	mm^3	0.55	RVE	Volume of veins with DR of 0 - 0.25 mm
Volume DR 2	mm^3	0.37	RVE	Volume of veins with DR of 0.25 - 0.8 mm
Volume DR 3	mm^3	0.27	RVE	Volume of veins with DR of above 0.8 mm
Width-to-depth ratio	-	0.55	RVE	Ratio of max. width to depth

Table S3: Petiole features Includes names, units, broad-sense clonal heritability estimates, and descriptions of the 18 traits related to petiole morphology and color. Abbreviations: avg: average, max: maximum, min: minimum. Note that Max. Feret is equivalent to petiole diameter that is used for validation in this work with real-world measurements.

**Feature**	**Units**	H^2	**Tool**	**Description**
Area	cm^2	0.49	Fiji	Total pixel count of petiole segmentation
Aspect ratio	-	0.41	Fiji	Ellipse major axis / Ellipse minor axis
Bottom blue	-	0.29	Fiji	Avg. blue value of petiole abaxial side
Bottom brightness	-	0.25	Fiji	Avg. brightness value of petiole abaxial side
Bottom green	-	0.27	Fiji	Avg. green value of petiole abaxial side
Bottom hue	-	0.15	Fiji	Avg. hue value of petiole abaxial side
Bottom red	-	0.22	Fiji	Avg. red value of petiole abaxial side
Bottom saturation	-	0.33	Fiji	Avg. saturation value of petiole abaxial side
Circularity	-	0.45	Fiji	$4\pi A/P^2$ where A: area and P: perimeter
Major axis length	cm	0.52	Fiji	Major axis length of the best-fit ellipse
Minor axis length	cm	0.20	Fiji	Minor axis length of the best-fit ellipse
Max. Feret	cm	0.55	Fiji	Max. distance between any two points in the petiole segmentation
Min. Feret	cm	0.09	Fiji	Min. distance between two parallel lines tangent to Max. Feret line
Perimeter	cm	0.55	Fiji	Sum of Euclidean distances between contour pixels in the petiole segmentation
Roundness	-	0.39	Fiji	$4A/(\pi M^2)$ where A: area, M: major axis
Solidity	-	0.10	Fiji	A/C where A: area and C: convex area
Volume	mm^3	0.43	RVE	Length multiplied by cross-section area estimated from petiole diameter
Width	cm	0.25	Custom	Avg. diameter of the center 20% of the petiole
